# Once Versus Thrice Daily Colistin in Critically Ill Ptients with Multi-Drug Resistant Infections

**Published:** 2017

**Authors:** Monireh Ghazaeian, Majid Mokhtari, Mehran Kouchek, MirMohammad Miri, Reza Goharani, Robabeh Ghodssi-Ghassemabadi, Mohammad Sistanizad

**Affiliations:** a *Department of Clinical Pharmacy, Faculty of pharmacy, Shahid Beheshti University of Medical Sciences, Tehran, Iran. *; b *Department of Pulmonary and Critical Care Medicine, Shahid Beheshti University of Medical Sciences, Tehran, Iran. Emam Hossein Medical and Educational Center, Shahid Beheshti University of Medical Sciences, Tehran, Iran. *; c *Department of Biostatistics, Faculty of Medical Sciences, Tarbiat Modares University, Tehran, Iran.*

**Keywords:** Procalcitonin, Colistin, Critically ill patients, Dosing interval, MDR infections

## Abstract

The aim of this study was to evaluate the procalcitonin (PCT) changes in two different high-dose colistin regimens in the treatment of multi-drug resistant MDR gram negative infections in ICU patients.

This is a prospective study of adult ICU patients with bacteremia and ventilator associated pneumonia (VAP) caused by MDR gram negative pathogens. Patients were assigned to two colistin administration groups. Group A received 9 and group B received 3 million international units every 24 and 8 h respectively. Baseline characteristics and measurements of PCT concentrations at the start, the 3^rd^ and the 5^th^ day of the antibiotic therapy and their trends between the two groups were recorded and compared.

of 40 patients enrolled, 34 completed the study protocol, of whom 30 (88.2%) had (VAP) and 4 (11.8%) had bacteremia. There were no statistically significant differences in the baseline characteristics between the two groups. The mean PCT levels in two study groups were; 2.34, 1.24, and 0.95 in group A and 5.89, 1.24 and 0.8 in group B at the baseline, 3^rd^ and 5^th^ day of colistin administration respectively (P=0.47). The ICU length of stay (LOS) in days and ICU mortality were; 31.31, 35.3% and 32.06, 22.2% in groups A and B (P=0.39, 0.87), respectively.

Conclusion: We did not find any statistically significant differences in the serum PCT levels, ICU LOS or ICU mortality, between the two groups, who received maximum recommended dose of CMS with 2 different intervals of every 8 or 24 h.

## Introduction

Inappropriate antibiotic therapy including wrong doses and/or duration of therapy in patients with intensive care unit (ICU) acquired infections, is associated with high mortality and morbidity which has been demonstrated in multiple studies (1-3).

On the other hand, excessive use of broad-spectrum antibiotics is associated with the development of resistant strains of different microorganisms, longer length of stay in ICU and hospital (1).

The increasing prevalence of infections caused by multi drug-resistant (MDR) gram-negative bacteria such as Pseudomonas *aeruginosa* and Acinetobacter baumannii have become a serious health problem worldwide (2). This has in turn led to the reintroduction of older antibiotics, such as colistin, with their potential adverse reactions and strong tendency for development of the newer resistant strains(3-5)

Colistin is a cationic polypeptide antimicrobial agent with a narrow bactericidal spectrum of activity against gram negative bacteria including *P.aeruginosa*, *Acinetobacter* spp. and Klebsiella spp.(6, 7). This agent is frequently administered in critically-ill patients suffering from (MDR) gram-negative infections (8-10). The available injectable formulation of colistin is penta-sodium colistin methane sulfonate or colistimethate sodium (CMS) which is an inactive prodrug with lesser potency and toxicity than colistin sulfate (11). 

In critically ill patients, the existing colistin dosing schedules may result in suboptimal peak levels corresponding to the (MIC) break points of MDR gram-negative bacteria, leading to inappropriate delays in effective management of these microorganisms. Consequently, different approaches such as administration of CMS in higher doses and using extended dosing-intervals, have been suggested to achieve a profile (12-17).

In order to guide antimicrobial administration more efficiently and to prevent their overuse, several serum biomarkers have been studied in different clinical settings. One such biomarker is procalcitonin (PCT) which has been studied extensively over the past two decades as a serum marker of systemic infection and sepsis.

Circulating procalcitonin is a peptide of 114 amino acids, lacking the N-terminal dipeptide alanine-proline. In addition to the calcium homeostasis, procalcitonin play pivotal roles in the metabolic and inflammatory host response to microbial infections.(18, 19)

Five systematic reviews have evaluated the role of PCT in managing antibiotic administration in critically ill patients (20-24). All of them come to a comparable deduction that PCT can reduce the duration of antibiotics use by around 2–3 days, without any significant effect on mortality or the rates of reinfection.

In a study, Crain *et al* demonstrated that employment of (PCT), as a tool for antibiotics de-escalation led to significant reduction (55%) of antibacterial use in patients with severe community-acquired pneumonia (25).Stolz *et al*. on the other hand showed that the rates of hospitalization due to acute exacerbations of chronic obstructive pulmonary disease was not significantly different between the (PCT) guided and control groups during 6 months follow up (26).

The aim of our study was to evaluate the PCT changes in the two different high-dose colistin regimens, used for the management of MDR gram negative infections in ICU patients. The rationale of our study was based upon the pharmacokinetics and pharmacodynamics of this agent, where its administration in the form of once daily infusion could have superior bactericidal properties, hence more favorable impact on PCT reduction in this patient group.

*Method*

This is a prospective, randomized-controlled trial performed in the general ICU of a 550 bed university hospital in Tehran, Iran, from 2014 to 2015. The Ethics Committee of the Shahid Beheshti University, Tehran, Iran approved the study protocol. The study was registered in Iranian Registry of Clinical Trials. (Registration date: July 10, 2014, Number: IRCT2014062510178N7).

Adult ICU patients with the diagnosis of bacteremia and ventilator associated pneumonia (VAP) with MDR gram negative organisms were included in the study. (VAP) was defined according to the criteria of the American Thoracic Society Consensus Conference on (VAP) (27) .Bacteremia was defined by microbial growth in one blood culture bottle (28). MDR organisms were defined based on the ECDC/CDC characterization as those organisms with resistance to at least one agent in three or more antimicrobial classes (29).

Colistin was prescribed as CMS (Colomycin; Forest Laboratories, United Kingdom) prepared in 100-mL sterile isotonic saline and was infused over 30 minutes. Using permuted box randomization, patients were randomly assigned to group A and B. Patients in group A received CMS 9 million IU once daily and patients in group B received CMS 3 million IU every 8 h.

Inclusion criteria were age >18 years, and documented MDR infections. The exclusion criteria were pregnancy, breastfeeding, body mass index of over 35 Kg/m^2^, and duration of colistin treatment less than 3 days. Serum PCT levels were measured at the start, 3^rd^ and 5^th^ days of the CMS therapy and their differences between the two dose intervals were compared. 

The measurements of PCT samples were made by electrochemiluminescence immunoassay using the COBAS e 411 equipment (Hoffmann-La Roche, Inc, Basel, Switzerland). A serum (PCT levels) of 0.1 µg/L or less indicated the lack of infection and led to the strong recommendation against antibiotics use. PCT levels of 0.1-0.25 µg/L suggested bacterial infection to be unlikely, 0.25- 0.5 µg /L suggested bacterial infection to be possible and 0.5 µg/L or greater strongly considered the presence of bacterial infection ((30, 31).

*Statistical analysis*

Continuous variables were expressed as mean ± SD if they were normally distributed and categorical variables were expressed as frequencies (percentages). Chi-square test, independent sample t-test, and repeated measure ANOVA were used for analysis. P-values less than 0.05 were considered statistically significant. All analyses were done using SPSS statistical software version 21 (IBM, Armonk, NY, USA).

## Results

We enrolled 40 patients in the study, 6 had to be excluded because CMS administration was less than 72 h (3 were on hemodialysis, one was transferred outside ICU and 2 expired before the 3^rd^ day of the study). Among 34 remaining patients who completed the study protocol, the dominant medical diagnosis was (VAP) in 30 cases (88.2%) and the remaining 4 had bacteremia (11.8%). There were no statistically significant differences in baseline characteristics between the two groups. (Table 1)

The PCT concentrations were measured in both groups at the baseline, day 3 and 5 after the administration of colistin and differences in total (CMS) exposure, according to resolution of clinical signs and decline in the (PCT) concentrations were compared in the two study groups. 

The serum (PCT) level changes were not significantly different between groups A and B of (CMS) dose interval groups during the study period (Table 2).

**Figure 1:**
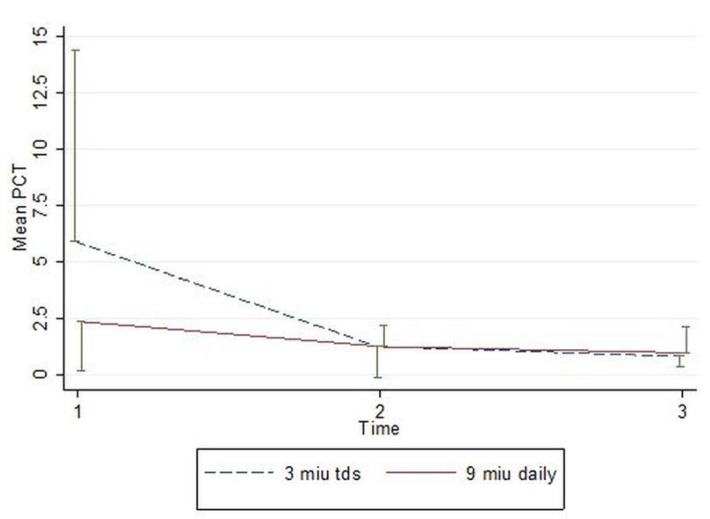
Mean (half error bar) of PCT of study groups.

In a two-way repeated measure (ANOVA) the PCT levels between two groups, for the corresponding days did not decrease significantly(p=0.468) and the reduction in the PCT levels throughout the study time was not statistically significant either (p=0.131). (Figure1).

The ICU LOS in groups A and B were, 31.31±10.8 and 32.06±14.1 (P=0.866) and ICU mortalities in groups A and B were, 6 (35.29%) and 4 (22.2%) (P=0.392), respectively.

## Discussion

The increase in the frequency of antimicrobial resistance is strongly determined by the choice of antibiotic agents, pattern of their use, infection prevention strategies and the microorganism characteristics. In the current era of the emergence of extensive and serious antibiotic resistance, we have remained with limited choices of anti-microbial agents for the management and reversal of this potentially fatal situation (32).

Reintroduction of colistin as an option for managing MDR gram-negative infections seemed inevitable (7, 8).However, continuing growth of gram negative bacterial resistance and inherent properties of colistin are two main challenging issues complicating determination of the appropriate dosing and intervals of this agent in different critical care settings. Several reports have demonstrated that even the administration of 9 million units per day of CMS, which is 50% or more over the usual recommended dose by the manufacturer, did not provide adequate serum levels for the concentration/MIC ratios needed to manage many MDR strains (12, 16).

In our study, we used colistin only for the treatment of severe infections resistant to other commonly used potent antibiotics. All patients in our study were infected by multi drug-resistant K. *pneumonia*, A. *baumannii* and P.*aeruginosa*. Based on the results of previous studies, we preferred to administer higher doses (9MIU per day) than the standard dose of colistin. 

Pharmacokinetic/pharmacodynamics studies have shown colistin to display concentration-dependent killing against gram negative organisms (33, 34)and high-dose and extended interval dosing regimens(13, 17) might be more efficacious in improving clinical outcome. on the contrary, there is some evidences against the extend interval regimen due to the concerns over emergence of resistance to colistin (35).

**Table 1:** Demographic and clinical Characteristics of patients

	**P - value**	**9 MIU Once Daily**	**3 MIU TDS**
Age	0.082	45.59 ± 18.3	55.71 ± 14.3
APACHEII	0.765	20.47 ± 5.1	20.47 ± 5.1
Length of stay	0.866	31.31 ± 10.8	32.06 ± 14.1
CMS duration	0.688	12.82 ± 4.3	13.53 ± 5.8
Sex (m/f)	0.999	(11/6)	(11/6)
**Source of infection**			
BSI, n (%) of cases	0.57	2(11.1%)	3(17.6%)
VAP, n (%) of cases		15(88.23)	14(82.4%)
**Combination with other antibiotics**			
Aminoglycosides		6	3
B-lactams		2	3
Quinolones		3	3

APACHE II: Acute Physiology and Chronic Health Evaluation; MIU: Million International Unit; VAP, ventilator-associated pneumonia; BSI, bloodstream infection

**Table 2:** PCT concentration and CMS duration

	**CMS groups**		
**P-value**	**9 MIU Once Daily**	**3 MIU TDS**	**Parameters**
			**PCT**
0.602	2.34 ± 3.9	5.89 ± 16.6	Baseline
0.056	1.24 ± 1.6	1.24 ± 2.6	Day 3
0.839	0.95 ± 2.03	0.80 ± 0.9	Day 5
0.369	14(10)	14 (4)	CMS duration*
0.866	31.31 ± 10.8	32.06 ± 14.1	ICU LOS^ǂ^
0.392	6 (35.29%)	4 (22.22%)	ICU mortality, n (%)

CMS: Colistin Methane Sulfonate; PCT: Procalcitonin, (ng/mL, Mean ±SD); LOS: Length of stay; Days (IQR: Interquartile Range).

* Median days (IQR).

In our study, (CMS) treatment was continued until the surveillance cultures detected that no organisms or clinical symptoms of patients were improved. We attempted to compare the effects of CMS administration in the extended interval of 9 MIU daily infusion (A) vs. 3 MIU every 8 h (B), on the serum PCT reduction trends, ICU LOS, and ICU mortality.

Although the baseline serum (PCT) values in group B (5.89µg/L) were higher than the values in group A (2.34 µg/L) and the rates of (PCT) reduction appeared to be numerically higher in the former group, neither of these values reached statistical significance. Trends in the ICU (LOS) were similar in both groups and although ICU mortality was higher, 6 vs. 4 in groups A compared to group B, these differences were not significant either. 

The limitations of our study were single center design and small sample size.

Larger and multicenter studies are required to further elucidate the biomarkers role in the optimization of colistin dosing regimens in order to improve clinical course of antibiotic therapy and to prevent the emergence of colistin resistance.
